# sHSPdb: a database for the analysis of small Heat Shock Proteins

**DOI:** 10.1186/s12870-016-0820-6

**Published:** 2016-06-13

**Authors:** Emmanuel Jaspard, Gilles Hunault

**Affiliations:** Université d’Angers, UMR 1345 IRHS, SFR 4207 QUASAV, Angers, France; INRA, UMR 1345 IRHS, Beaucouzé, France; Agrocampus-Ouest, UMR 1345 IRHS, Angers, France; Université d’Angers, Laboratoire d’Hémodynamique, Interaction Fibrose et Invasivité tumorale hépatique, UPRES 3859, IFR 132, F-49045 Angers, France

**Keywords:** Small heat shock proteins, Amino acids sequence, Physico-chemical properties

## Abstract

**Background:**

small Heat Shock Proteins (sHSP) is a wide proteins family. SHSP are found in all kingdoms and they play critical roles in plant stress tolerance mechanisms (as well as in pathogenic microorganisms and are implicated in human diseases).

**Results:**

sHSPdb (small Heat Shock Proteins database) is an integrated resource containing non-redundant, full-length and curated sequences of sHSP, classified on the basis of amino acids motifs and physico-chemical properties. sHSPdb gathers data about sHSP defined by various databases (Uniprot, PFAM, CDD, InterPro). It provides a browser interface for retrieving information from the whole database and a search interface using various criteria for retrieving a refined subset of entries. Physicochemical properties, amino acid composition and combinations are calculated for each entry. sHSPdb provides automatic statistical analysis of all sHSP properties. Among various possibilities, sHSPdb allows BLAST searches, alignment of selected sequences and submission of sequences.

**Conclusions:**

sHSPdb is a new database containing information about sHSP from all kingdoms. sHSPdb provides a classification of sHSP, as well as tools and data for the analysis of the structure - function relationships of sHSP. Data are mainly related to various physico-chemical properties of the amino acids sequences of sHSP. sHSPdb is accessible at http://forge.info.univ-angers.fr/~gh/Shspdb/index.php.

## Background

Heat Shock Proteins (HSP) are proteins whose expression is increased when cells are exposed to elevated temperatures or other stress. Among HSP, sHSP belong to the superfamily of protein chaperones. They counteract the irreversible aggregation of misfolded proteins. sHSP are ubiquitous proteins found in all living organisms, from bacteria to mammals and especially in plants [1–4]. Moreover, sHSP display a variety of sub-cellular localization and/or tissue distribution [3, 5].

sHSP contain : (i) a highly variable (in both length and in sequence) N-terminal domain; (ii) a very conserved central domain called the Alpha Crystallin Domain (ACD) involved in dimerization leading to the building block of higher order sHSP structure; (iii) a short C-terminal domain that links monomers inside dimmers [6–8]. A study of all sHSP merged have shown that the average length of ACD is 90 ± 10 residues [9] and this length is much more constant than the N- and C-terminal domains. However, we have classified sHSP into 21 classes and this value of average length of ACD does not apply anymore when considering sHSP class by class. The N-terminal part of ACD is likely not necessary for dimerization or chaperone activity, but seems required for higher order aggregates formation [10].

As members of the chaperone network, sHSP play important roles in cells exposed to heat and other stress, but their functional molecular mechanisms are not fully elucidated. sHSP monomers (12–42 kDa) usually associate into large homo-oligomers of mostly 24 subunits [9, 11]. sHSP quaternary structures have a high degree of plasticity due to changes in their oligomeric state under different cellular conditions or to exchange of their subunits. Loosening of subunit organization leads to more dynamic properties thus enhancing the available chaperone sites for the client proteins. For example, it was shown that the sequence RLFDQ (found at the N-terminal part of ACD of HSP27 - class number 12) contributes to the higher order assembly of their subunits as well as their structural stability [12]. Structural studies of numerous sHSP have brought details in monomer, transition of the oligomeric state [13–15] and sHSP chaperone mechanism [12, 16].

However, such approaches do not allow the comparison of more than a few sHSP at a time. On the contrary, sHSPdb offers access to large-scale analysis of sHSP. Moreover, since sHSP is a very large protein family there is a wide discrepancy in annotation, definition and terminology: sHSPdb provides a classification of sHSP based on selective motifs, physicochemical properties and previous classification [17–19]. sHSPdb has been conceived with multiple functions to search and describe the different sHSP. sHSPdb provides a global vision of sHSP physicochemical properties, amino acid usage, literature as well as statistical analysis on any sHSP dataset built by the user. Such a resource will be invaluable for computational analyses of sHSP structure - function relationships, especially regarding the specific roles of each of the three domains. sHSPdb is useful to gather information about sHSPs from all kingdom. sHSPdb provides the largest set of sHSP sequences, a new classification and a precise description of each class of sHSP. Physico-chemical properties and statistical analysis provided by sHSPdb represent an actual plus-value.

## Construction and content

### Data sources and characteristics of the dataset

To fill sHSPdb, we used a two-stage process. The first step filled automatically the fields of the tables of the database using PHP and perl scripts, getting the information from text and XML files from the following databases: NCBI/Proteins, NCBI/CDD, NCBI/Taxonomy, EBI/Picr, UNIPROT, Interpro, SANGER/Pfam, AMIGO. Starting with the NCBI accession number, we retrieved the « *GenPept* » file to derive the GI number, the eventual PFAM, CDD and Interpro [8, 20, 21] identifiers and the textual information. Then, using the cross-references of EBI/Picr we obtained the Uniprot accession number and name to complete and double-check the data. The main sources to fill sHSPdb are information contained in « *GenPept* » files and in the corresponding Uniprot files. The primary request to get « *GenPept* » files from the public database NCBI contained a construction of Boolean and keywords. This allowed the retrieving of ≈ 17,300 « *GenPept* » files stored in sHSPdb. Nevertheless, there was a very high redundancy of sequences for many organisms. The second step was a manual check to ensure that all information and links were pertinent and relevant to the sHSP issue. The pertinence of the files annotation and that of the sequences were checked using: (i) the sHSPdb « *Search* » option (see below); (ii) BLAST homology analysis and multiple alignments software; (iii) MOCAR (http://forge.info.univ-angers.fr/~gh/wstat/Mocar/), a program developed specifically for our sHSP classification based on pattern motif recognition in any Fasta sequences dataset; (iv) the « *Analyze* » and the « *Statistical analysis* » tools implemented in sHSPdb; (v) Finally, the motifs determined for each class of sHSP were further used to perform PHI-Blast allowing to get additional sequences.

Sequence conflict (in particular in the case of AGI entries for *Arabidopsis thaliana*) was checked using annotations from the Uniprot files. Identical sequences were removed using a program developed for sHSPdb feeding. This allowed detecting truncated, ambiguous (e.g., annotated « unnamed protein », « hypothetical », « unknown product »…, although some few populated sHSP classes may contain such annotated files) or false sHSP sequences. Sequences containing the undefined amino acid symbol (X) were removed. All files are stored in sHSPdb but only non-redundant, full-length and annotated ones are accessible.

The two holding chaperones (holdases) families, i.e., HSP31 and HSP33, cannot be considered formally as sHSP since they do not harbor the ACD domain, and interact with client proteins through Cys and disulfide bonds formation [22]. However, these redox regulated molecular chaperones have physico-chemical properties close to those of sHSP and also contribute to protein homeostasis under stress conditions. Moreover, they are redox regulated molecular chaperones: they protect both thermally unfolded and oxidatively damaged proteins from irreversible aggregation and play an important role in the bacterial defense system toward oxidative stress. Therefore, the two classes HSP31 and HSP33 were incorporated into sHSPdb.

The main goal of sHSPdb is the analysis of the structure-function relationships of sHSP. This is why only amino acids sequences are implemented. Today the user can nevertheless BLASTX any nucleotide sequence against sHSPdb to check the existence of similar or homologous sHSP.

Finally, sHSPdb contains ≈ 4800 curated, non-redundant and full-length sHSP sequences. More than 3200 sequences (67 %) are classified into 21 classes on the basis of their corresponding unique sequence motif. Roughly 400 additional sequences are classified on the basis of various criteria, eye inspection and our expertise. The remaining unclassified sHSP sequences have been assigned to a class 99 with ≈ 530 of these sequences being annotated Hsp20. Even if we expect to which class sequences of class 99 belong, they are not yet classified because they do not match exactly the regular expression of any of the defined motifs. Since there is no program available to automatically determine such complex discriminants motifs for any set of sequences, we are currently developing appropriate methods to address this question. In particular, we are using the recent constraint programming approach, based on a two-stage program whose last stage reduces to the classical minimum set covering problem, though, as for now, the complete and « perfect » characterization of all classes is not achieved. Partials results have already been published [23, 24].

Sequences are mainly issued from ≈ 2355 Bacteria, ≈ 2259 Eukaryota (among which ≈ 1050 Viridiplantae) and ≈ 129 Archaea (Table 1).

**Table 1:** Main taxonomy of the organisms in sHSPdb. Only groups containing more than 10 sequences are indicated

129 Archaea	2355 Bacteria	2259 Eukaryota	
21 Crenarchaeota 77 Euryarchaeota 31 Thaumarchaeota	198 Actinobacteria 11 Aquificae 95 Bacteroidetes 12 Chlamydiae 22 Chloroflexi 111 Cyanobacteria 14 Deinococcus 279 Firmicutes 15 Planctomycetes 1501 Proteobacteria 39 Spirochaetes 14 Thermotogae	1180 Opisthokonta 1051 Viridiplantae	
		Opisthokonta	Viridiplantae
		203 Fungi 977 Metazoa	12 Funariacae 49 Pinales 285 Liliopsida 687 Eudicotyledons

sHSPdb contains also ≈ 14,240 non-accessible entries (i.e., not retained after our curation). This dataset is important when the database is updated and for further analyses of sHSPdb since it may serve for statistical studies and forbid wrong entries in the curated set.

### Organization and main features of sHSPdb

A link to a guided tour is proposed in the homepage to learn how to use the interface of sHSPdb. This guided tour proposes various scenarii of search. The user can select any entries through various parameters and conduct further analyses using the implemented tools. For this purpose, sHSPdb has three main features: (i) the browse mode that allows the user to consult all or part of the database; (ii) the search mode based upon multiple search criteria; (iii) the export mode to retrieve sequences in different formats.

#### The browse mode

**Fig. 1:**
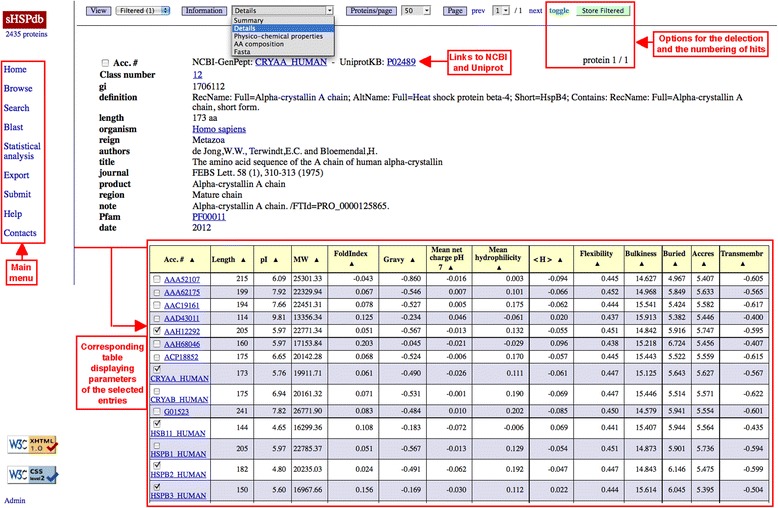
Browse interface for accessing the whole sHSPdb content. The « Search » mode provides an advanced search with multiple parameters and permits retrieval of very fine subsets of data. The user can also retrieve sequences by entering amino acids motif either exact or degenerated using regular expressions with sophisticated syntax. Among other information, the « Summary » option provides links to general databases, the name of the sHSP and its putative function. The « Details » option provides more information. A series of physicochemical properties are given by selecting the « Physicochemical properties » option. The « AA comp » option displays the amino acid composition of sHSP. Finally, the « Fasta » option provides all sequences in FASTA format

It allows consulting the whole database (Fig. 1). The « *Summary* » option provides the NCBI-GenPept accession number and the Uniprot accession number, the name of the sequence and of the organism, the putative function of the sHSP (if any). The « *Details* » option provides more information (up to 20 fields from the GenPept or the Uniprot files). The accession numbers, the name of the organism, the PFAM, the CDD and the InterPro numbers provide a link to their relevant website. A series of physicochemical properties (length/pI/MW/FoldIndex/GRAVY/mean net charge at pH7/mean hydrophilicity/mean hydrophobicity < H>/flexibility/bulkiness/accessibility of residues/transmembrane tendancy of residues) are given by selecting the « *Physicochemical properties* » option. The « *AA comp* » option displays the amino acid composition of sHSP. Finally, the « *Fasta* » option provides all sequences in Fasta format. The « *Align sequences* » option allows to align selected sequences.

#### The search mode (multiple search capabilities)

It corresponds to an advanced search with multiple parameters and permits retrieval of very fine subsets of data (example of search: « sHSP of the PF00011 family from *Arabidopsis thaliana*, containing the motif of Class 1, associated to the key word « chromosome », published by an author called Walker in 2012, with a length comprised between 150 and 200 amino acids »). The search can be made by using a sophisticated text expression accepting wildcards and Booleans - this search applies to all fields of the whole database; by accession number (accepting wildcards) - one or multiple accession number(s) separated by a space can be searched at one time; by organism; by PFAM or CDD numbers; by date; by range of length of amino acids sequence. The user can also retrieve sequences by entering amino acids motif either exact or degenerated using regular expressions with sophisticated syntax, allowing to specify complex motifs. The known motifs (Table 2) implemented in sHSPdb result from our analysis.

**Table 2:** sHSP classification in sHSPdb

sHSP class (Usual name)	Localization (plants sHSP)	Examples of representant and/or annotation	Motif allowing to classify sequences^a^	Motif position in sequences	Number of sHSP classified^b^	Main taxonomy
Class 1 (Class I)	Cytosol-nucleus	TaHsp16.9 PsHsp18.1	[AGPSTV][ADEGHMNQTV][ILPSVY][KR][AFGNPQST][IV][ADEHKNQ][IFV][ACGNSTY][ADEGV]?(.{1,6})?$	C-terminal	362	Viridiplantae
Class 2 (Class II)	Cytosol-nucleus	17–17.9 kDa HSP	[ILNV].{0,1}[KNR].{0,1}[PT]P[APQ].{1,2}[AKR].{0,6}[IV].{0,5}$	C-terminal	111	Viridiplantae
Class 3 (Class_IV)	Endoplasmic reticulum		WH.{8,10}[FS][IW]R[QR][FL].{2,2}P.{3,3}[^K]	Central	68	Viridiplantae
Class 4 (Class_V)			[^E]A[RS]AA$	C-terminal	46	Viridiplantae
Class 5 (Class_VI)		21.7–22.3 kDa	D.{2,3}D.{1,2}PLW	N-terminal	64	Viridiplantae
Class 6 (Class M)	Mitochondria	HSP23	[LM].{10,10}[KR].{1,2}[DEKQ][DEKNQ][DEGHPQ].{2,2}[DEN].{3,3}[IV].[^H][DENQ]$	C-terminal	118	Viridiplantae
Class 7 (Class P)	Plastids	HSP26 to HSP26.8 CPsHSP21	[DEFHNQSTY][AGKPRST][KT][IV][FHILMTV][DEG][IRV][DEHKNQS][ILV][EKNQR]G?$	C-terminal	102	Viridiplantae
Class 8 (Class Px)	Peroxysome		[IV].{3,3}[^D][KR]L$	C-terminal	52	Viridiplantae
Class 9 (Class RA)	Ribosome Associated	sHSP15 RNA-binding S4	P[^P]K.{2,2}R[QR].{7,27}$	C-terminal	247	Bacteria
Class 10 (HSP20/IbpA/IbpB)		Hsp20 - IbpA/B - HspA/B - HspB6	P[GKP][FHMY][DN][ILV]	N-terminal	545	Bacteria Fungi
Class 11 (HSP20/HspC)			[FILMV][^P].?[DEQ][FILMPV][ADEKNQS][^S][FILMV][FLVW][ADEGHIMNQS]	N-terminal	679	Bacteria Archaea Fungi
Class 12 (HSP27 /aA-crystallin /aB-crystallin)		HspB1 aA or aB -crystallin HspB4 or 5	[AGHNPQRSTY][DHKR][AFILMPV].[DES][DQ].F[AG]	N-terminal	534	Metazoa Bacteria
Class 13 (beta-9)			[LM][LP][SV].{2,2}L.[ADEN].{11,14}F[KQ]	N-terminal	56	Metazoa
Class 14 (HSP Beta_3)			PVRY[EKQ]	N-terminal	84	Metazoa
Class 15 (HSP Beta_7)			EIKI$	C-terminal	26	Metazoa
Class 16 (HSP Beta_11)		HSP family B	[ADE]TFW	N-terminal	76	Metazoa
Class 17 (HspQ)			K[FY][AFG][IL]G	N-terminal	88	Bacteria
Class 18 (HSP42)		Hsp42p	DEEL.{1,16}$	C-terminal	16	Fungi
Class 19 (HSP30)			[PST][AEIV][HLR][PST][LH]W[PT][AEHT]	N-terminal	24	Metazoa
Class 20 (HSP31)		HchA « Holdases »	TG.{4,4}E.{11,11}G	N-terminal	147	Bacteria Fungi
Class 21 (HSP33)		33 kDa chaperonin « Holdases »	C.[FY]C.{3,3}Y	C-terminal	135	Bacteria

^a^Sequences classified via the motif of the class. Regular expression syntax for amino acid motifs: [XYZ] means X or Y or Z; X? means X is optional; [^XY] means not X nor Y; $ means C-terminal position;.means any amino acid; X{n,m} means n to m times X. ^b^Number of sequences retrieved in sHSPdb using the motif indicated

The user can perform a similarity search by using BLASTP implemented in sHSPdb. This allows obtaining additional information and retrieving the best scoring sequences through the classical BLAST output interface. It is also possible to BLASTX a nucleotide sequence against sHSPdb to check the existence of similar or homologous sHSP in order, for example, to construct a putative sHSP cDNA sequence from EST.

Many options are proposed for the output: (i) when entries have been retrieved, the information can be displayed in any of the views of the browse mode and the fields such as accession number, taxonomy, PFAM, CDD and InterPro numbers are linked to their original web site according to the relevant information found at the NCBI; (ii) the user can select or deselect any entry resulting from his search (« *Toggle* » option) and the final selection can be stored. This is true for all displays (browse mode, BLAST output, …); (iii) one original point is the re-ordering (ascending or descending sort) of data displayed in tables, for a better comparison and analysis of data, using any shown information as the sort criterion; (iv) the user can automatically align selected sequences.

#### The export mode

All sequences in SHSPdb can be exported trough the « *Export* » interface. The « *View* » menu displays the entries in the order of the last actions made by the user (all, filtered, selected or blasted). The « *Format* » menu displays three exporting formats: FASTA, XML and Excel (CSV).

#### The analyze tool

One original feature of sHSPdb is the possibility to obtain numerous information of any self-built dataset using the left panel. The right panel allows selecting the desired information. For categorical data, results are sorted tables of counts, respectively with respect to alphabetic order and values of counts; for quantitative data, classical descriptive statistics and graphics (such as means, median, histograms…) are provided.

#### The statistical analysis tool

**Fig. 2:**
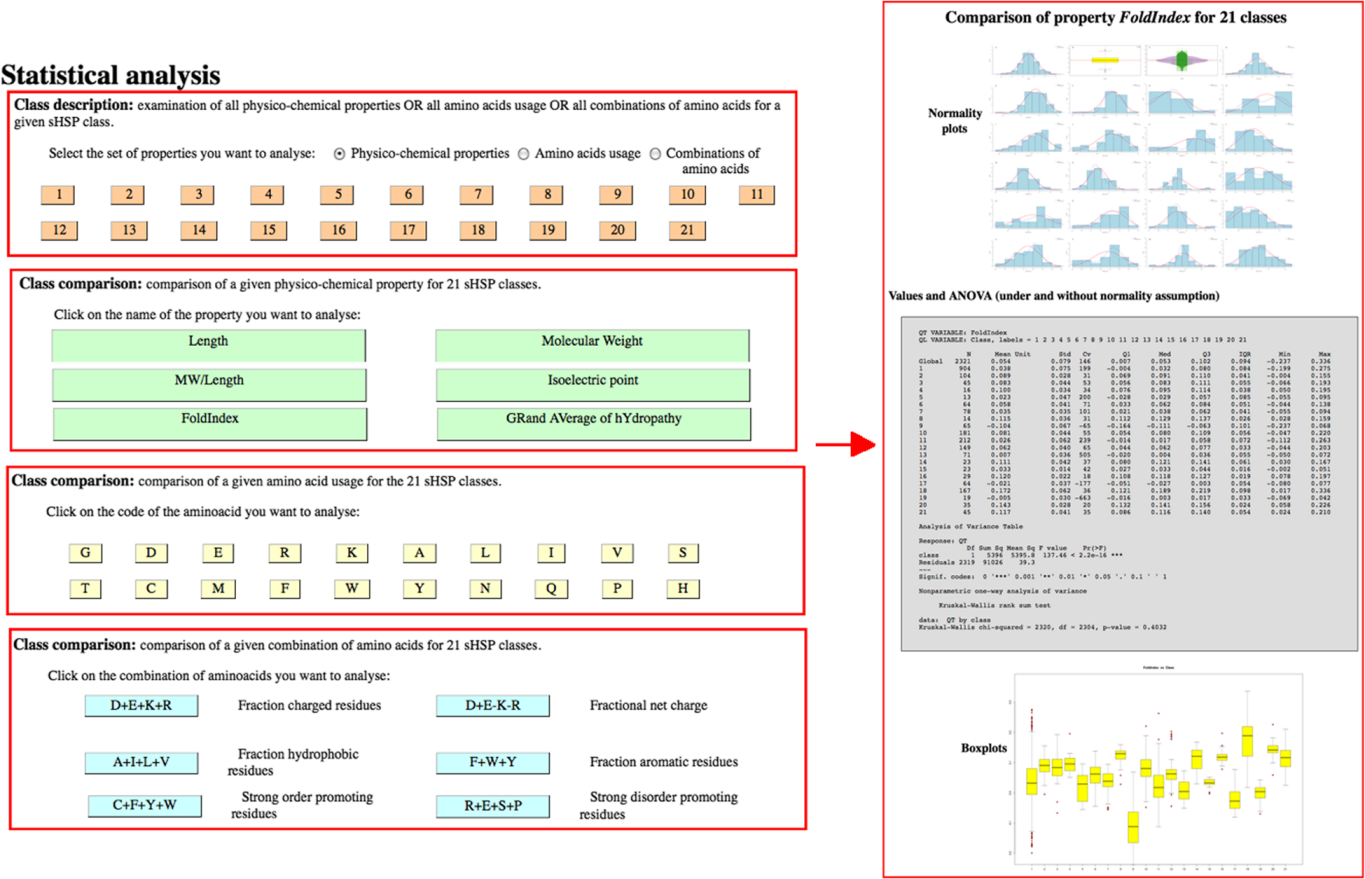
The statistical analysis tool. One original feature of sHSPdb is the possibility to dynamically generate plots of physico-chemical properties or amino acids usage of sHSP using different criteria. Therefore, one can immediately make multiple comparisons of self datasets. The user can access to statistical data through the menu « *Statistical analysis* ». This option displays « *Class description* » (examination of all physico-chemical properties or all amino acids usage or all combinations of amino acids for a given sHSP class) or « *Class comparison* » (comparison of a given physico-chemical property for the sHSP classes). Plots are automatically generated and are clickable to be resized

Another original feature of sHSPdb is the possibility to dynamically generate plots of physico-chemical properties or amino acids usage of sHSP using different criteria. Therefore, one can immediately make multiple comparisons of self datasets. The user can access to statistical data through the menu « *Statistical analysis* ». This option displays « *Class description* » (examination of all physico-chemical properties or all amino acids usage or all combinations of amino acids for a given sHSP class) or « *Class comparison* » (comparison of a given physico-chemical property for the sHSP classes). Plots are automatically generated and are clickable to be resized (Fig. 2). The statistical analysis includes both descriptive (means, medians…) and inferential (parametric or non parametric ANOVA) computations and graphics (histograms with density and normal approximation, boxplots, beanplots when accurate…).

#### The submission form

The user can submit sequence(s) through a submission form. After verification of their relevance, they will be integrated in sHSPdb.

### Database implementation

sHSPdb is a fast, interactive, platform independent Web-based database with a user-friendly interface written in PHP. Some options need Javascript to be activated. Information is provided to the user from a MySQL relational database. The statistical computations are performed using the R software called by a PHP script.

## Utility and discussion

### sHSPdb versus other sHSP datasets

Poulain et al. [9] reported about a dataset including 3787 sHSP sequences. However, this dataset contains some redundancy: 774 sequences from this dataset were aligned and 70 sequences are identical, corresponding to a redundancy of 9 %. It can be thus estimated that the entire dataset correspond roughly to 3400 different sHSP, i.e., less than the number of sHSP contained in sHSPdb. Moreover, this dataset is limited to a flat PDF file with accession numbers and thus does not provide any details or functionalities. Our selection is very stringent since we excluded most of all files annotated « putative », « probable », « unknown », « hypothetical », « unnamed protein product », « synthetic construct », … in order to increase the quality of annotation even if less sequences were retained. We did not consider fragments, i.e., files annotated « partial ». Sequences with such annotation but additional indication and/or classifiable on the basis of a motif were retained.

To our knowledge, there is only one other database dedicated to HSP (HSPIR: http://pdslab.biochem.iisc.ernet.in/hspir/shsp.php) which gather accession numbers and FASTA files for HSP protein sequences, and provides general information about the structure and function of the different families of HSPs [25]. However, HSPIR contains only ≈ 1300 sequences of sHSP, and offers access to only basic search and BLAST tools. sHSPdb appears thus as a unique comprehensive and highly implemented database for the computational and statistical analysis of sHSP.

### Classification of sHSP

sHSP constitute a very large protein sub-divised family of proteins. However, there is a wide discrepancy in annotation, definition and terminology. sHSPdb provides a unified and homogeneous classification of sHSP. One of the most important parameter for the sHSP classification is the consensus motif discriminating each class from another (Table 2). The proposed classification in classes is based on the expertise brought by sHSPdb, literature data and previous classifications [1, 2, 9, 17, 19, 26–30].

The quite simple C-terminal motif [ADHKNQ]G.[AILV] matches ≈ 89 % of the sequences and a more sophisticated central motif [APS][EGM].{4,5}[ADENQSK] matches ≈ 99 % of the sequences. Such motifs therefore describe almost all sHSP and are therefore not pertinent for discrimination. Since most of sHSP classes contain the ACD (with exception of sHSP classes 9, 16, 17 and 21), we searched selective motifs either in the N-terminal or the C-terminal domains, if any (Table 3).

**Table 3:** Some length characteristics of sHSP and « alpha-crystalin-unrelated sHSPs »

sHSP class	Sequences length range (min - max)	Motif position^a^	Mean limit values of domains length		
			N-terminal	ACD^b^	C-terminal
Class 1	130–282	132–273	1–50	51–141	142–157
Class 2	129–175	131–151	1–49	50–139	140–156
Class 3	165–328	90–255	1–72	73–164	165–198
Class 4	119–211	115–207	1–42	43–123	124–148
Class 5	172–254	41–118	1–86	87–182	183–194
Class 6	101–279	185–212	1–120	121–202	203–218
Class 7	157–266	215–229	1–129	130–222	223–235
Class 8	115–183	109–177	1–28	29–127	128–144
Class 9	100–175	98–127	*NA* ^c^	*NA*	*NA*
Class 10	120–498	8–200	1–40	41–137	138–160
Class 11	111–356	6–109	1–51	52–139	140–156
Class 12	108–374	14–214	1–59	60–151	152–176
Class 13	146–277	26–152	1–53	54–142	143–167
Class 14	141–187	13–54	1–63	64–145	146–152
Class 15	108–262	105–259	1–79	80–160	161–178
Class 16	114–230	34–80	*NA*	*NA*	*NA*
Class 17	102–131	5–6	*NA*	*NA*	*NA*
Class 18	332–453	317–440	1–256	257–349	350–384
Class 19	191–220	6–26	1–81	82–165	166–211
Class 20	133–298	54–163	1–42	43–133	134–156
Class 21	161–344	136–320	*NA*	*NA*	*NA*

^a^Range of starting position of the motif among all sequences of the class. ^b^Alpha Crystallin Domain. ^c^Not applicable since these HSP do not contain ACD and are defined as « alpha-crystallin-unrelated sHSPs »

This motifs search underlined the difficulties with annotation. For example, the motif [AFLSTVWY]P[AGPS][FHY][DN][ILV] matches ≈ 540 sequences whose annotations are mainly « heat shock protein HSP20, « HSP20 family protein » or « Hsp20/alpha crystallin family protein ». Therefore, the high selectivity of our motifs helps to ameliorate global annotation such as the generic « HSP20 ».

Previous classifications were first based on the intracellular localization of the sHSP: cytoplasmic-nuclear compartment (classes CI, CII and CIII), plastids (class P), endoplasmic reticulum (class ER), mitochondria (class M), peroxisomal (class Po or Px). These seven plant sHSP classes are to date the best characterized of the plant sHSP (they are coded by 14 genes in *Arabidopsis thaliana*). The complete analysis of *Arabidopsis thaliana* genome led to four additional cytoplasmic-nuclear sHSP and a second unique family of sHSP targeted to the mitochondria. Thus, higher plants may contain at least 12 conserved sHSP subfamilies. Organelle-targeted sHSP are so far specific of plants, with some exceptions (mitochondria-targeted sHSP from *Drosophila melanogaster* and from *Toxoplasma gondii*) [31, 32]. However, it remains difficult to ascertain the sub-cellular localization of proteins, especially on large numbers of proteins because predictors are not reliable enough. Finally, the taxonomy of organism among each class is homogeneous indicating that our classification of sHSP is accurate (Table 2).

**Fig. 3:**
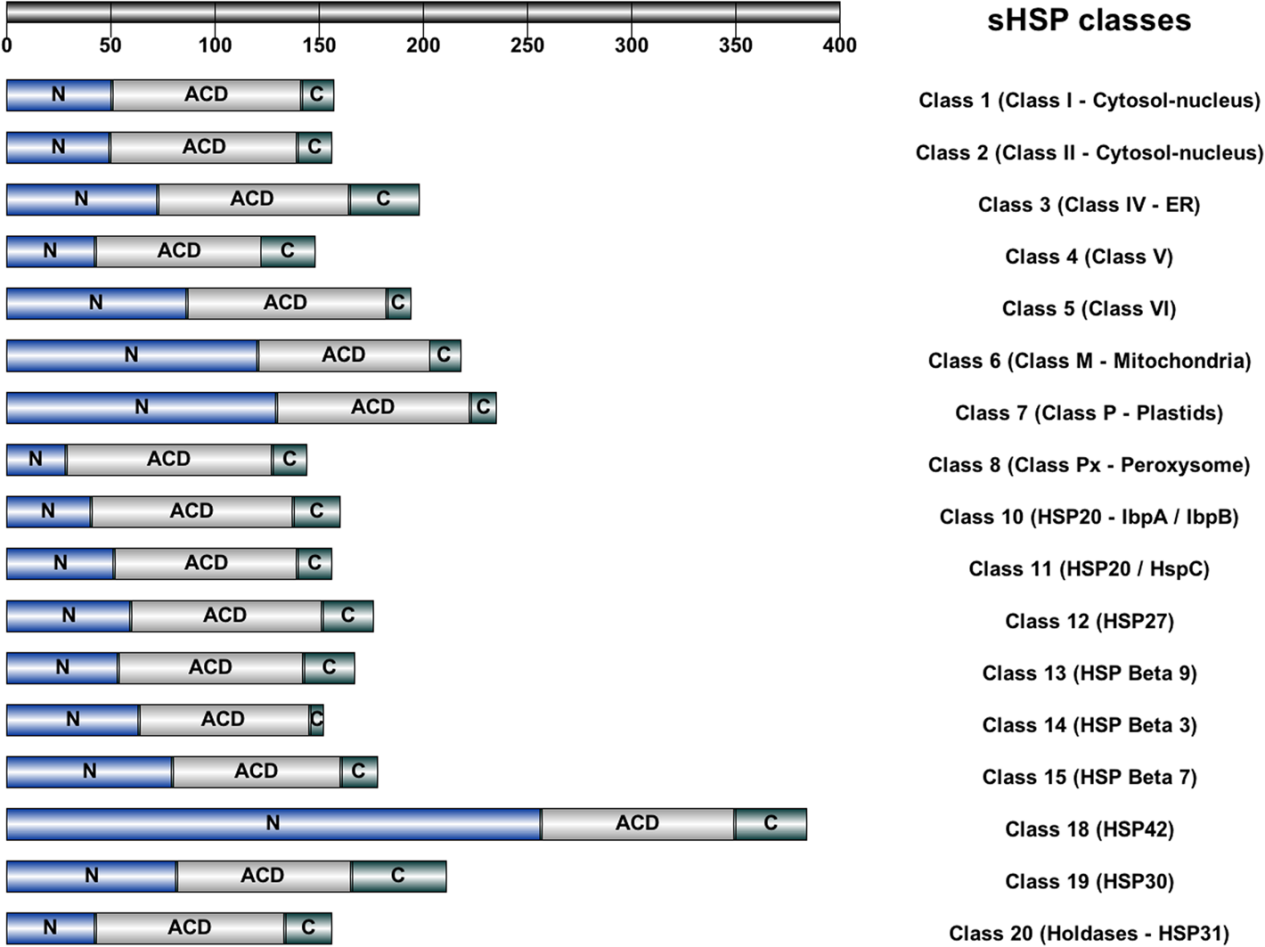
Comparison of the mean value of the size of the N-terminal domain, ACD and the C-terminal domain. Only HSP classes containing an ACD are represented (see Table 3). The upper scale indicates the size of the sequences in amino acids. ER: endoplasmic reticulum

Such classification together with the statistical data automatically generated is thus useful. For example, sHSPdb help for analyzing the respective role of each of the three domains characterizing sHSP in oligomerization and substrate binding on a very large scale. A conserved motif, [ILV].[ILV], located in the C-terminal domain is critical for oligomer formation through contacts with a hydrophobic patch in ACD [9, 13, 33]. The ACD domain is an emblematic signature of sHSP in the literature [19, 28] and it is indeed found in 17 of the 21 classes in sHSPdb. Table 3 and Fig. 3 show the comparison of the mean value of the size of the N-terminal domain, ACD and the C-terminal domain.

Nevertheless, some heat shock proteins lacking an ACD sequence should be in our opinion considered as sHSP and defined as « alpha-crystallin unrelated sHSP ». Therefore, we have implemented 4 classes of sHSP without ACD. This classes of sHSP devoid of the ACD domain fall well within the molecular weight expected for sHSP. Their implementation provides additional information and allows more comparisons and analyses of sHSP.

### Short example of use of sHSPdb: analysis of mitochondrial sHSP

**Fig. 4:**
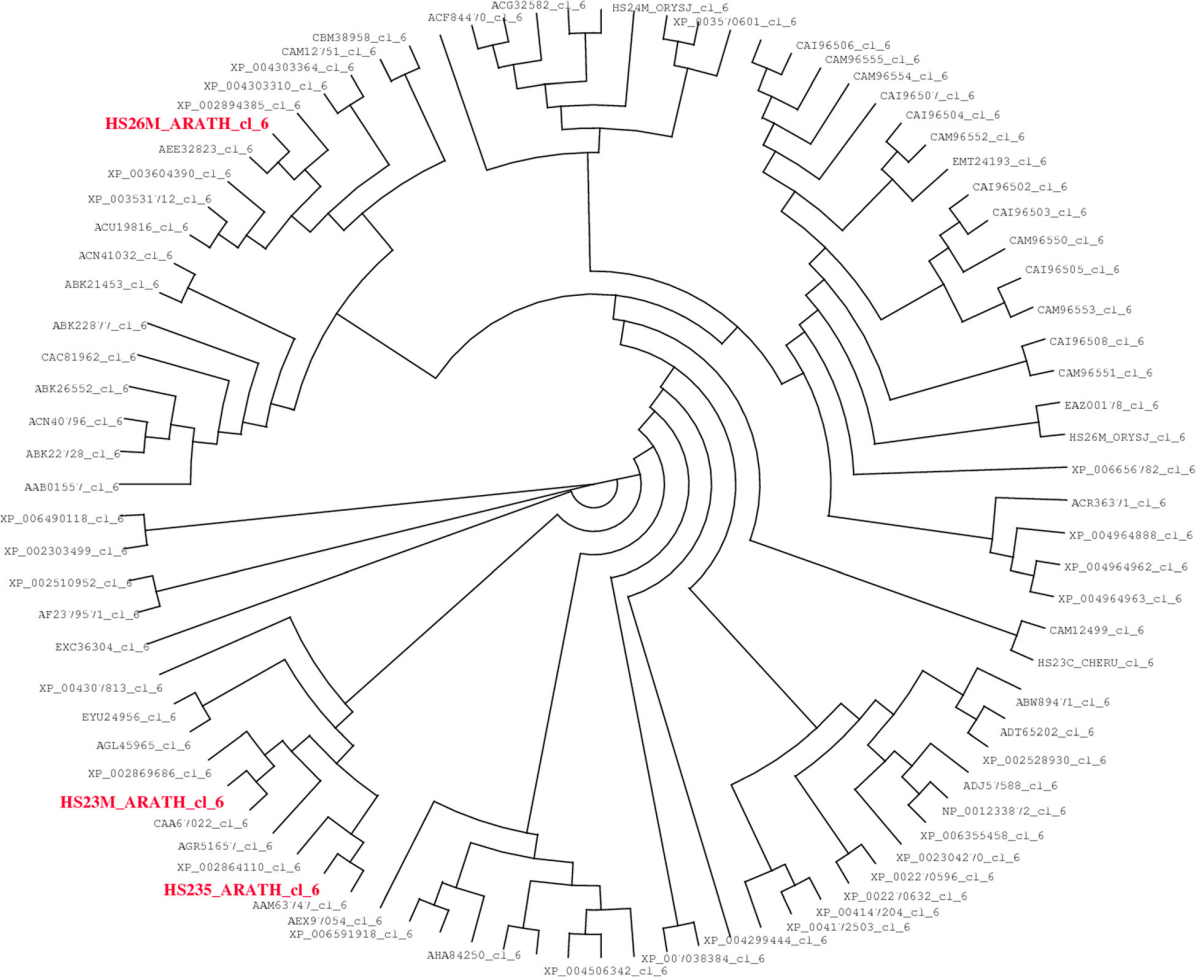
Phylogenetic tree of plant mitochondrial sHSP. 91 sequences of mitochondrial sHSP from 47 plant organisms were analyzed. The characteristics of the sequences are: a length from 187 to 248 amino acids; an isoelectric point from 4.620 to 10.15; a net charge at pH 7 from *−* 0.041 to +0.031; a FoldIndex from *−* 0.044 to 0.138; a hydropathy (GRAVY) from *−* 0.908 to *−* 0.274. The alignment was made using Clustal-W and the figure was drawn using Dendroscope [36]

**Fig. 5:**
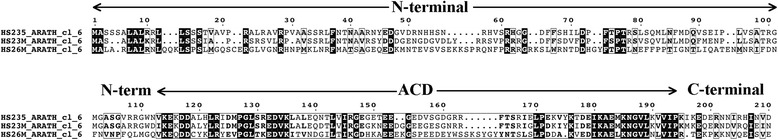
Alignment of three mitochondrial sHSP from *Arabidopsis thaliana*. The alignment was made using Multalin [37] and the figure was drawn using ESPript [38]

A phylogenetic tree of plants mitochondrial sHSP (class 6) has been built (Fig. 4). Five sequences correspond to mitochondrial sHSP from Arabidopsis thaliana (AAM63747, CAA67022, HS235_ARATH, HS23M_ARATH and HS26M_ARATH, all from class 6). The differences between AAM63747 and HS235_ARATH are [S/A]31 and [D/H]69 and the difference between CAA67022 and HS23M_ARATH is [D/H]143. The sequences of ACD and C-terminal domain are very similar (Fig. 5). The sequences of the N-terminal domain of HS235_ARATH and HS23M_ARATH are different from that of HS26M_ARATH and are more intrinsically disordered (negative FoldIndex for HS26M_ARATH). This may have a possible incidence on the dimer formation [34, 35].

## Conclusions

sHSPdb harbors a comprehensive dataset available for sHSP, together with tools designed for their online analysis. To our knowledge, there is no equivalent database for sHSP. sHSP are classified into classes on the basis of various parameters, especially on the basis of amino acids motifs that discriminate the classes. sHSPdb thus constitute an efficient tool: (i) for the compilation and the organization of growing data concerning sHSP; (ii) for the classification of the various sub-families of sHSP; (iii) for the design of experiments to elucidate the function of this important proteins; (iv) to help the analysis of the sHSP structure-function relationships.

Future developments and perspectives: (i) sHSP physico-chemical properties and sHSP amino acids usage are statistically analyzed for all sHSP classes. We will thus be able to compare the three domains (i.e., the N-terminal, the ACD and the C-terminal), thus bringing additional information to those already determined by structural methodologies. (ii) We are currently developing software for the analysis of sequence submitted by the users in order to predict if it belongs to any of the sHSP classes. (iii) Since deciphering the molecular functions of sHSP is a major issue, we will provide lexical tools (dictionaries by alphabetic order or occurrence or synonyms…) for a better semantic analysis of the words that describe the known elements of the function of sHSP. (iv) As previously noted, retained proteins that are not fully classified are under study with the help of some predicting values and of a constraint programming software under development.

## Availability and requirements

sHSPdb is a free database and visualization tool open to all users with no login requirements and can be accessed at the following URL: http://forge.info.univ-angers.fr/~gh/Shspdb/index.php. The web tool is functional on all modern web browsing environments including Mozilla Firefox, Safari and Google Chrome.

## Abbreviations

ACD, alpha crystallin domain; ER, endoplasmic reticulum; NA, not applicable; sHSP, small heat shock proteins; sHSPdb, small heat shock proteins database
